# Supplementary material for: Touchless interactive teaching of soft robots through flexible bimodal sensory interfaces
Source: Nat Commun. 2022 Aug 26;13:5030. doi: 10.1038/s41467-022-32702-5 (PMC9412806; doi:10.1038/s41467-022-32702-5)
Supplement: Supplementary file 1 — Supplementary Information [file 41467_2022_32702_MOESM1_ESM.pdf]

Supplementary Information for

Interactive teaching of soft robots through flexible touchless and tactile bimodal sensory interfaces

Authors:

*Wenbo Liu[1,†], Youning Duo[1,†], Jiaqi Liu[1,†], Feiyang Yuan[1†], Lei Li[1], Luchen Li[1], Gang Wang[1], Bohan Chen[1], Siqi Wang[1], Hui Yang[2], Yuchen Liu[3], Yanru Mo[3], Yun Wang[1], Bin Fang [4], Fuchun Sun [4], Xilun Ding[1], Chi Zhang[5,6], Li Wen[1]**

Affiliations:

[1] School of Mechanical Engineering and Automation, Beihang University, Beijing 100191, China.

[2] Institute of Semiconductors, Guangdong Academy of Sciences, Guangdong 510075, China.

[3] School of General Engineering, Beihang University, Beijing 100191, China.

[4] Tsinghua National Laboratory for Information Science and Technology, Department of Computer Science and Technology, Tsinghua University, Beijing, 100084, China.

[5] CAS Center for Excellence in Nanoscience, Beijing Key Laboratory of Micro-Nano Energy and Sensor, Beijing Institute of Nanoenergy and Nanosystems, Chinese Academy of Sciences, Beijing 101400, China

[6] School of Nanoscience and Technology, University of Chinese Academy of Sciences, Beijing 100049, China

† These authors contributed equally

* Corresponding author. Email: liwen@buaa.edu.cn

This file includes:

Supplementary Figures

Supplementary Figure 1: FBSS output signals as a finger approaches and presses it.

Supplementary Figure 2: Surface charge production on the external object and FBSS.

Supplementary Figure 3: Measurement system of the FBSS.

Supplementary Figure 4: The dynamic response of the FBSS.

Supplementary Figure 5: The signal-to-noise ratio (SNR) of the FBSS.

Supplementary Figure 6: The resolution of the FBSS.

Supplementary Figure 7: The FBSS integrated with a soft gripper.

Supplementary Figure 8: Comparing the driven air pressures during teaching and repeating in two-dimensional space.

Supplementary Figure 9: A human user teaching the soft manipulator to overcome an obstacle via the intelligent interactive interface.

Supplementary Figure 10: Comparing the driven air pressures during teaching and repeating in three-dimensional space.

Supplementary Figure 11: The trajectories of both teaching and repeating for multiple motion modes of the soft manipulator interacting with a single FBSS.

Supplementary Figure 12: Teaching experiments with multiple participants.

Supplementary Figure 13: Schematic diagram of quickly switching the position of a sensor on a soft manipulator.

Supplementary Figure 14: Comparing the driven air pressures during teaching and repeating while taking a throat swab.

Supplementary Figure 15: Fabrication processes of the flexible electrode layer and the flexible dielectric layer.

Supplementary Figure 16: Fabrication process of the liquid metal patch.

Supplementary Figure 17: Fabrication process of the soft manipulator.

Supplementary Figure 18: Chamber lengths of the bending segments with different pressure.

Supplementary Tables

Supplementary Table 1. Algorithm for touchless human-soft manipulator single segmentary interaction.

Supplementary Table 2. Algorithm for touchless human-soft manipulator interaction in two-dimensional space.

Supplementary Table 3. Algorithm for touchless human-soft manipulator interaction in three-dimensional space.

Supplementary Table 4. Comparison list of bimodal sensors.

Supplementary Movies

Supplementary Movie 1. The FBSS detecting a human finger approaching and pressing it.

Supplementary Movie 2. The FBSS detecting a tennis ball falling.

Supplementary Movie 3. The FBSS detecting a feather falling.

Supplementary Movie 4. A human finger controls LEDs via the FBSS.

Supplementary Movie 5. A human user controlling the soft manipulator via the FBSS.

Supplementary Movie 6. A self-reacting soft origami robot with the FBSS detecting and grasping a toy bug in sand.

Supplementary Movie 7. The soft gripper detecting and grasping a plastic cylinder with the FBSS.

Supplementary Movie 8. The interactive teaching and repeating processes of the soft manipulator with variable steps.

Supplementary Movie 9. Interactively teaching and repeating to grasp objects in different positions.

Supplementary Movie 10. The soft manipulator teaching process replayed at different speeds.

Supplementary Movie 11. Interactively teaching and repeating while overcoming an obstacle.

Supplementary Movie 12. Interactively teaching and repeating in three dimensions (front view).

Supplementary Movie 13. Interactively teaching and repeating in three dimensions (side view).

Supplementary Movie 14. Interactively teaching and repeating to grasp objects in different positions in three dimensions.

Supplementary Movie 15. Interactively teaching and repeating multiple motions by switching the positions of a FBSS.

Supplementary Movie 16. Interactively teaching two-dimensional movements using the "shifting sensors and teaching" method.

Supplementary Movie 17. Interactively teaching three-dimensional movements using the "shifting sensors and teaching" method.

Supplementary Movie 18. Repeating three-dimensional movements using the "shifting sensors and teaching" method.

Supplementary Movie 19. Interactively teaching and repeating to complete a pen-and-paper maze.

Supplementary Movie 20. Interactively teaching and repeating to take a throat swab.

Supplementary Movie 21. Interactively teaching of the soft manipulator to cross the obstacle and grasp a flower.

Supplementary Movie 22. Repeating of the soft manipulator to cross the obstacle and grasp a flower.

Text

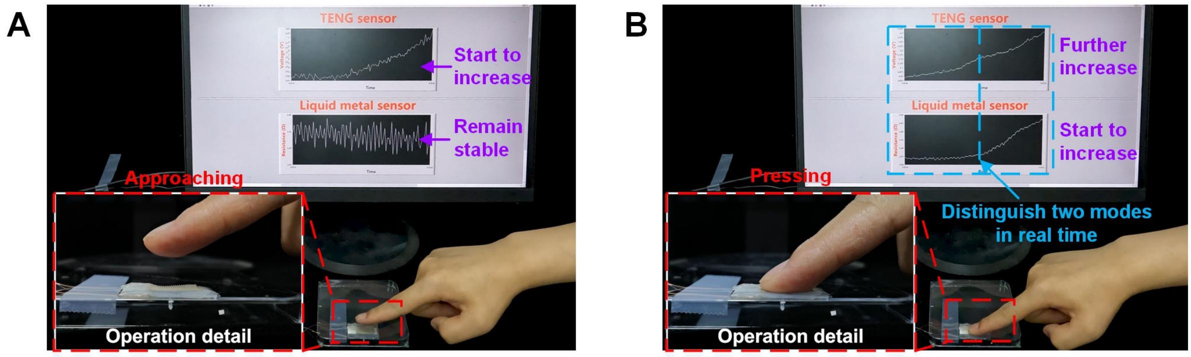

Supplementary Figure 1: FBSS output signals as a finger approaches and presses it.

(A) The output touchless signal increases as the finger approaches the FBSS, while the output tactile signal is negligible. **(B)** As the finger presses on the FBSS, the output touchless signal increases further and the output tactile signal starts to increase. It is hard to distinguish between the touchless and tactile modes by the output signal of the triboelectric nanogenerator alone. However, the FBSS based on a triboelectric nanogenerator sensor and a liquid metal sensor can transduce both tactile and touchless stimulations simultaneously and distinguish between the two modes in real time.

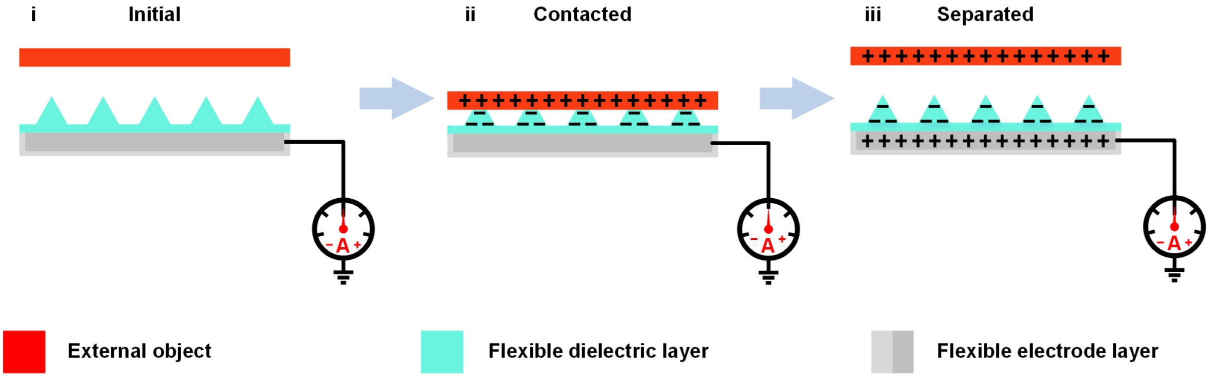

Supplementary Figure 2: Surface charge production on the external object and FBSS.

In the initial state (i), there is no charge on the surface of the external object (red) and flexible dielectric layer (cyan). In the second state (ii), equal negative and positive charges are generated on the flexible dielectric layer (cyan) and external object (red) from different electron affinities after a few repeated contacts. In the third state (iii), the external object (red) separates from the flexible dielectric layer (cyan) and these surface charges can remain for a long time.

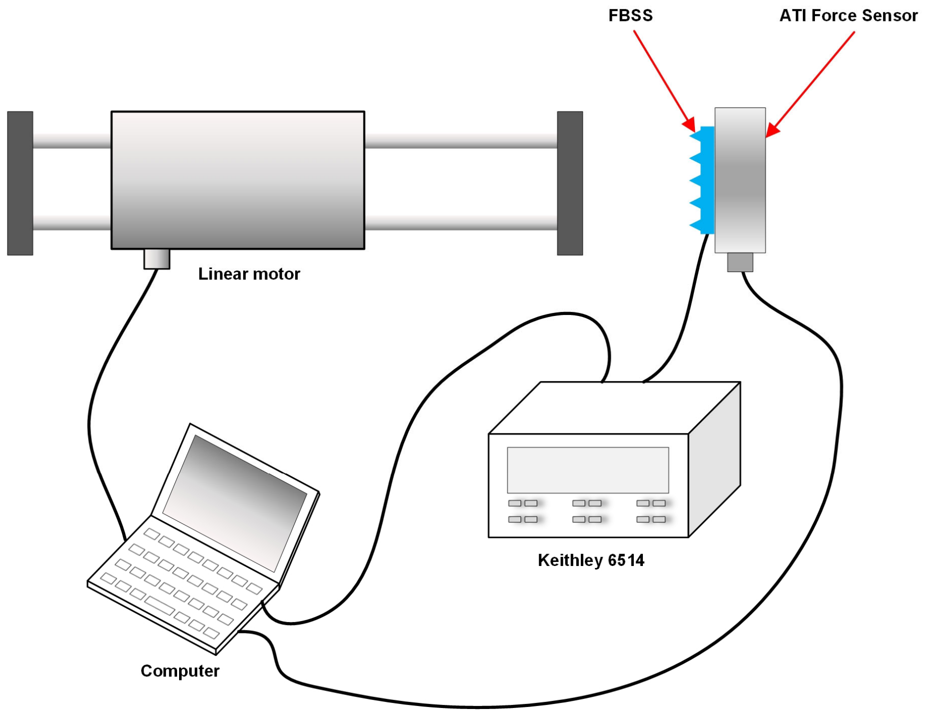

Supplementary Figure 3: Measurement system of the FBSS.

The FBSS is affixed to a force gauge (ATI Industrial Automation, mini40) and external object is attached to the end of a linear motor (LinMot, E1100). While the distance to and contact pressure on the FBSS was controlled by a computer (via the linear motor), the output voltage was measured with a 6514 electrometer and the resistance change of the FBSS was tested with a synchronous data acquisition card (National Instruments, USB-6356).

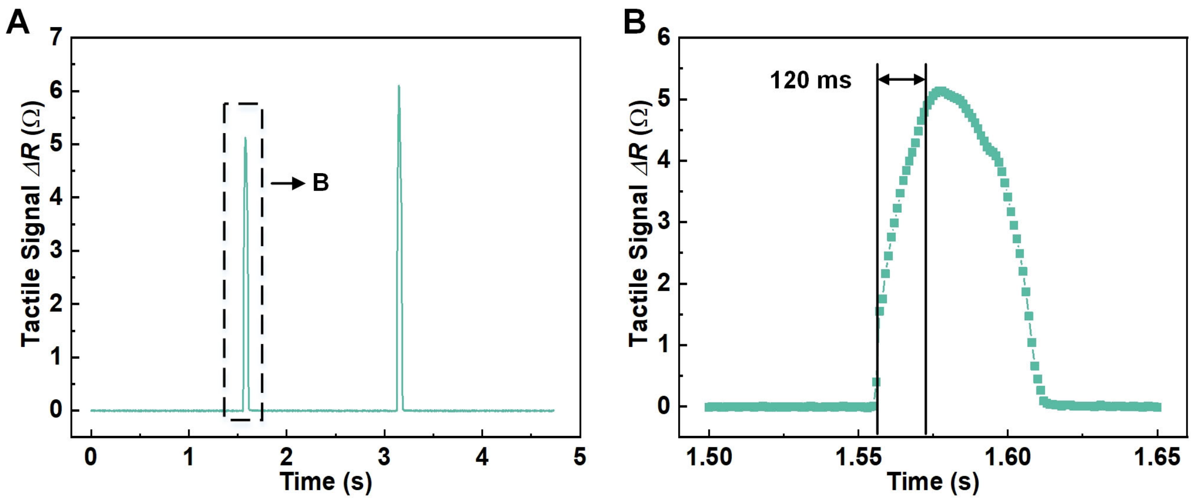

Supplementary Figure 4: The dynamic response of the FBSS.

(A) The experimentally measured change of the resistance of the FBSS under a fast pressure stimulus. **(B)** A close-up of the area indicated within the dashed box in **(A)**.

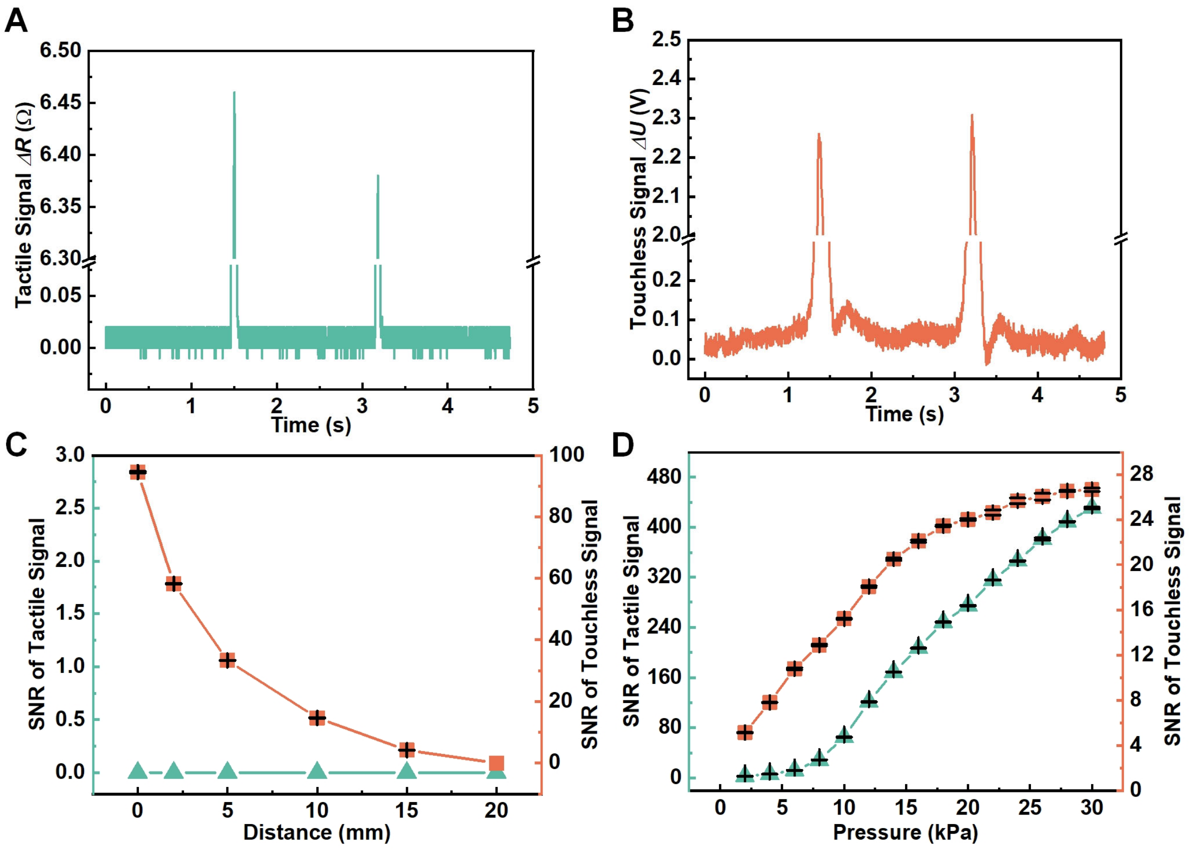

Supplementary Figure 5: The signal-to-noise ratio (SNR) of the FBSS.

(A), (B) The noise of tactile and touchless signals. **(C), (D)** The SNRs of the touchless and tactile signals. Error bars represent standard deviation, n = 5 independent replicates.

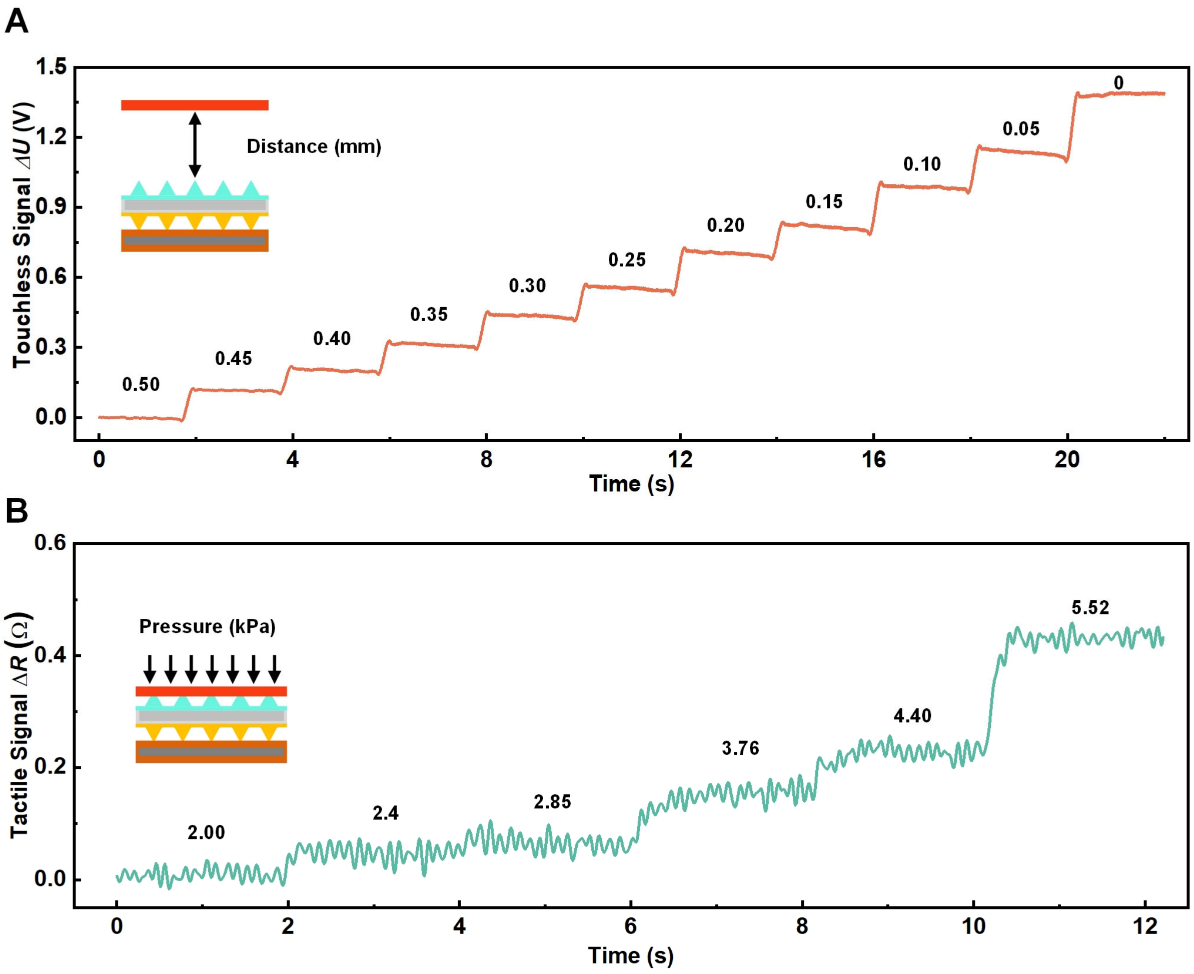

Supplementary Figure 6: The resolution of the FBSS.

(A) The resolution of touchless sensing. The distance between the testing surface (glass) and the FBSS gradually decreases from 0.5 to 0 mm. **(B)** The resolution of tactile sensing. The indenting pressure imposed on the sensor gradually increase from 2.00 to 5.52 N.

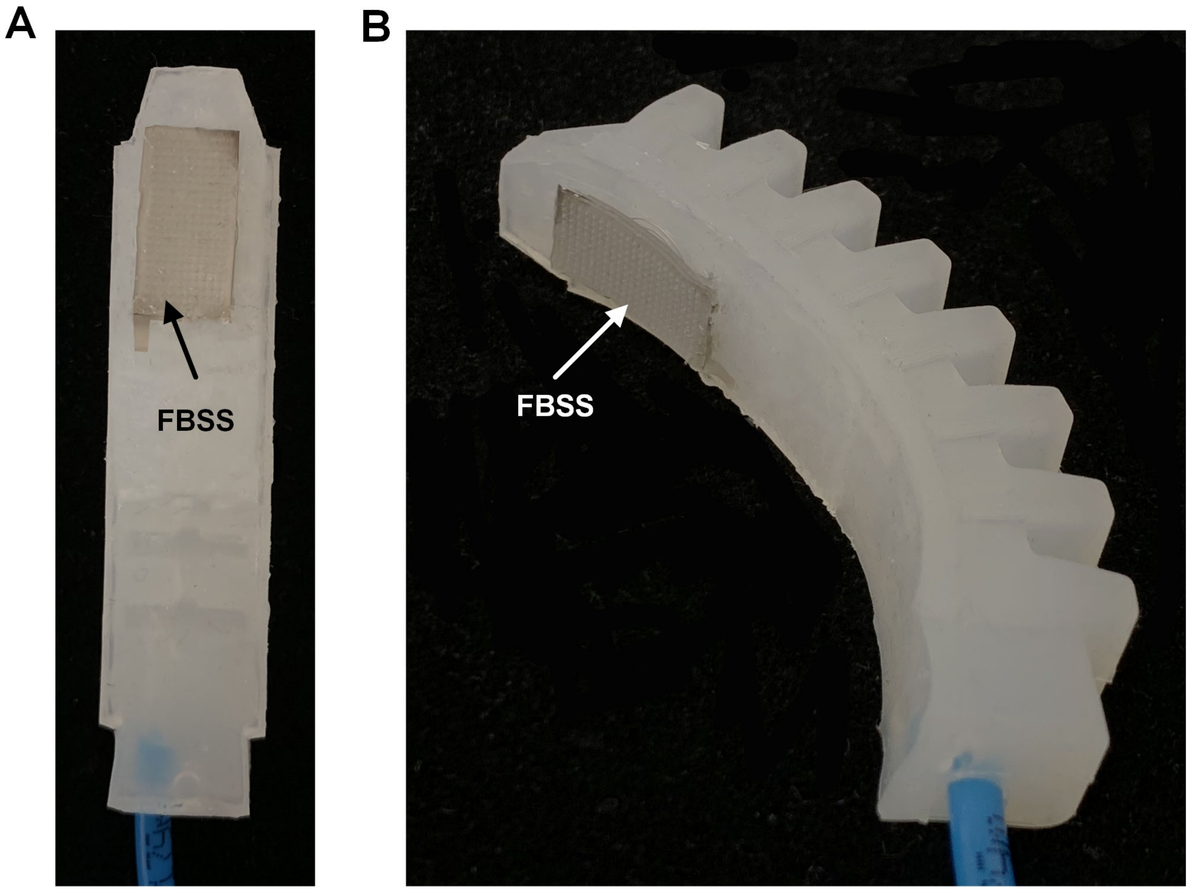

Supplementary Figure 7: The FBSS integrated with a soft gripper.

(A) The bottom of the soft gripper. **(B)** The soft gripper is actuated by air pressure.

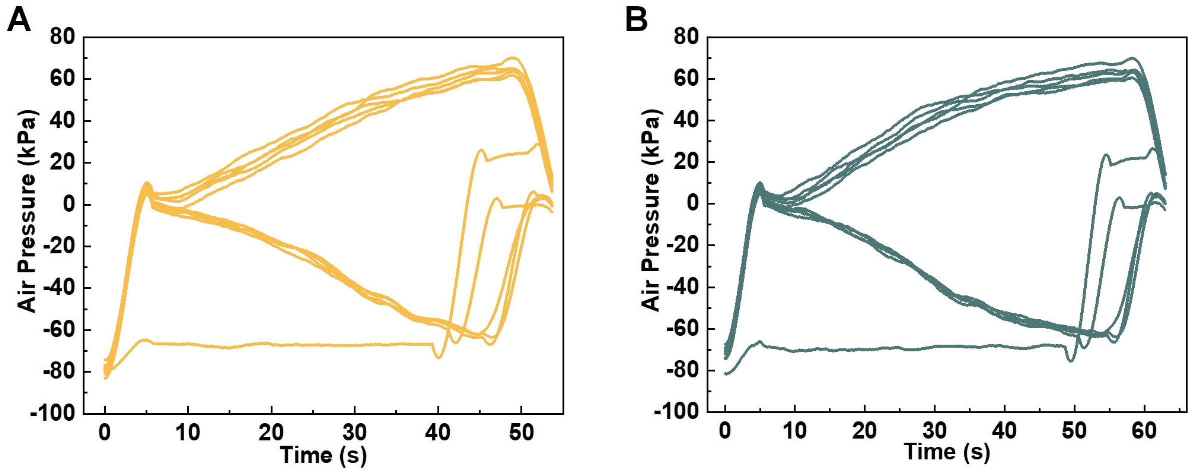

Supplementary Figure 8: Comparing the driven air pressures during teaching and repeating in two-dimensional space.

(A) Driven air pressure over time during teaching and **(B)** the same chart while repeating.

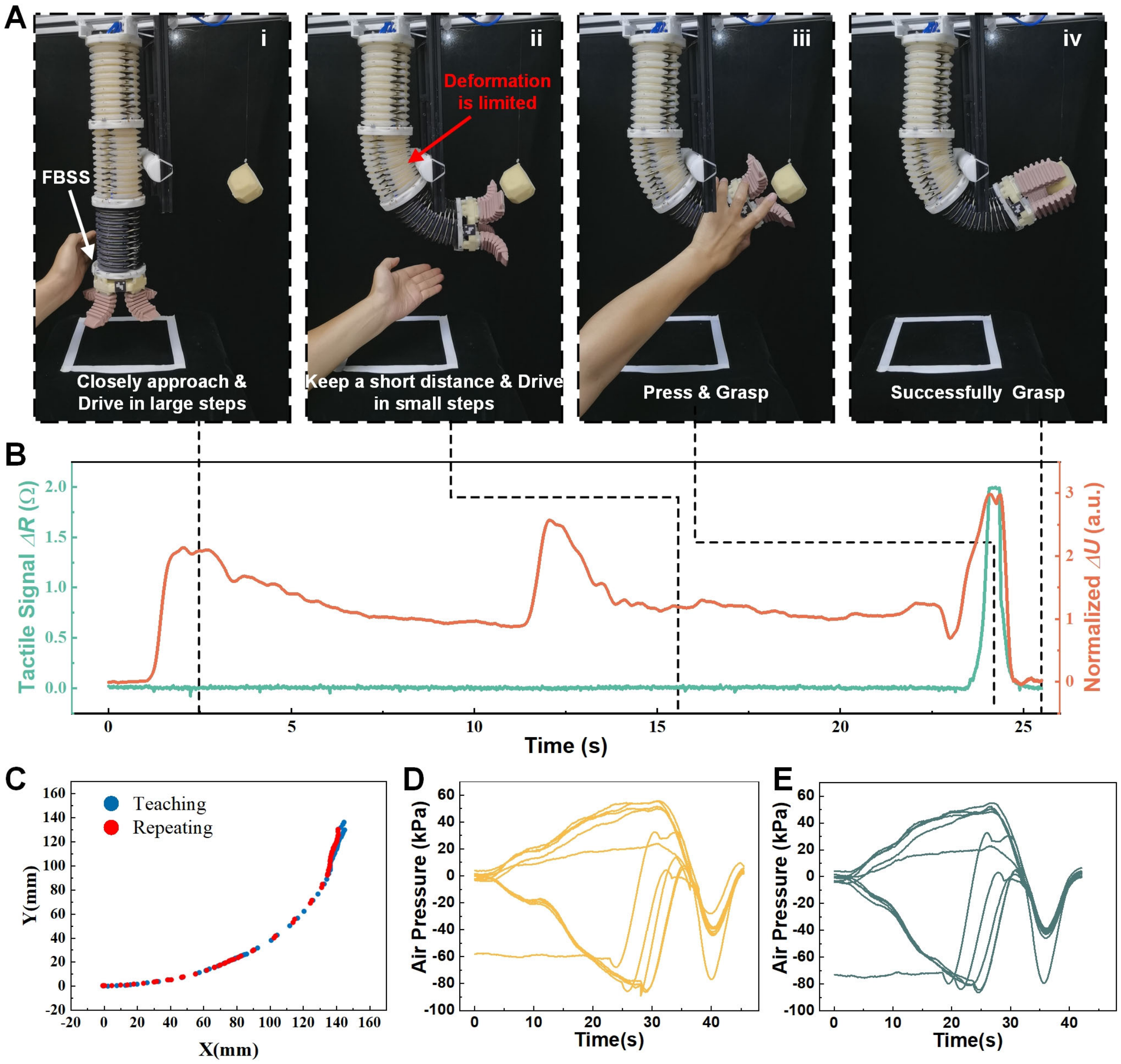

Supplementary Figure 9: A human user teaching the soft manipulator to overcome an obstacle via the intelligent interactive interface.

(A), **(B)** Photographs and signal curves during the teaching process. The whole teaching process can be divided into four stages. In stage (i), a human user touchlessly controls the soft manipulator with a hand by approaching the manipulator in large steps. In stage (ii), to precisely adjust the position of the soft manipulator, the user uses small steps. In stage (iii), the user instructs the soft gripper to grab the target by pressing the FBSS. In stage (iv), the manipulator grabs the target successfully and returns to its original position. **(C)** A comparison of spatial trajectories during teaching and repeating. **(D)**, **(E)** A comparison of the driven air pressures over time between the teaching and repeating processes.

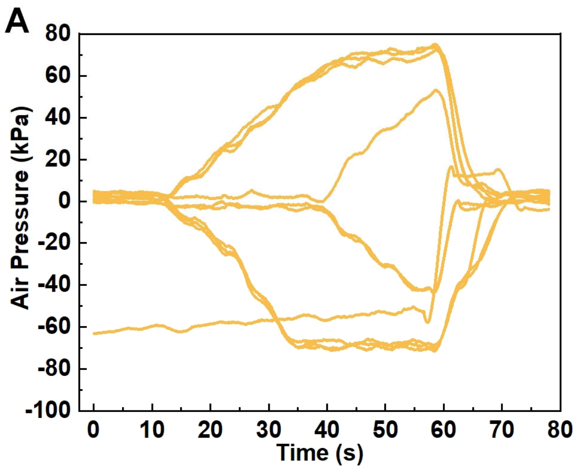

Supplementary Figure 10: Comparing the driven air pressures during teaching and repeating in three-dimensional space.

(A) Driven air pressure over time during teaching and **(B)** the same chart while repeating.

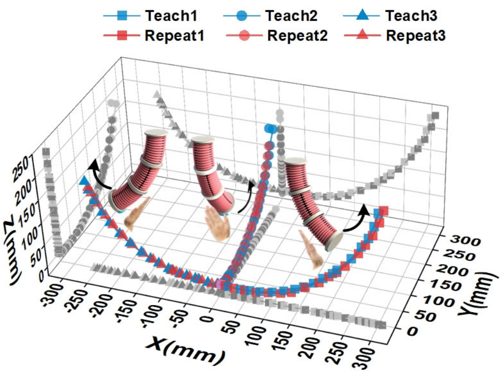

Supplementary Figure 11: The trajectories of both teaching and repeating for multiple motion modes of the soft manipulator interacting with a single FBSS.

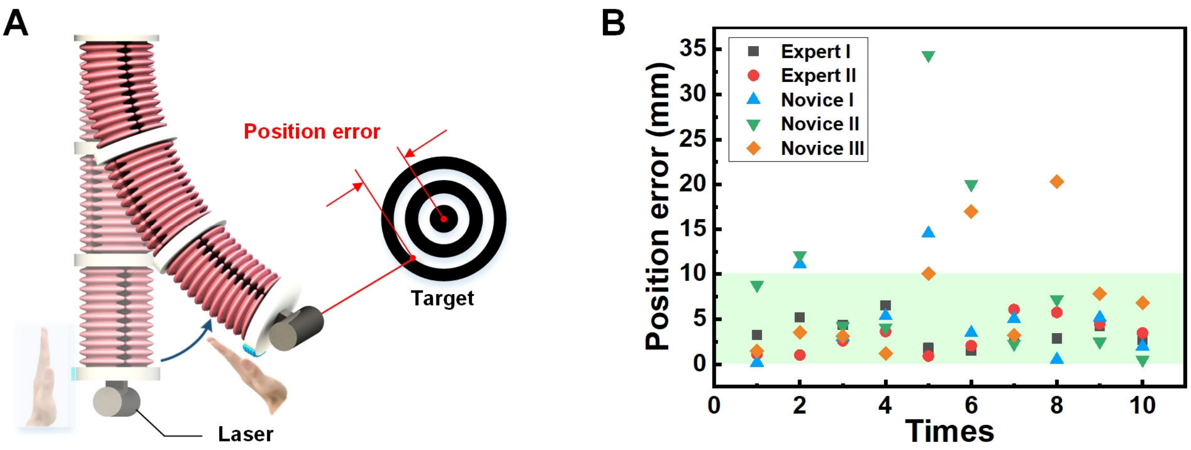

Supplementary Figure 12: Teaching experiment with multiple participants.

(A) The principle of measuring the position error in experiments. A laser pointer is attached to the soft manipulator and participants can control the position of the laser point by touchlessly teaching the soft manipulator. The position error is defined as the distance between the final position of the laser and the center of the target. **(B)** The positioning error of the manipulator after being taught by multiple human subjects, including two experts and three novices.

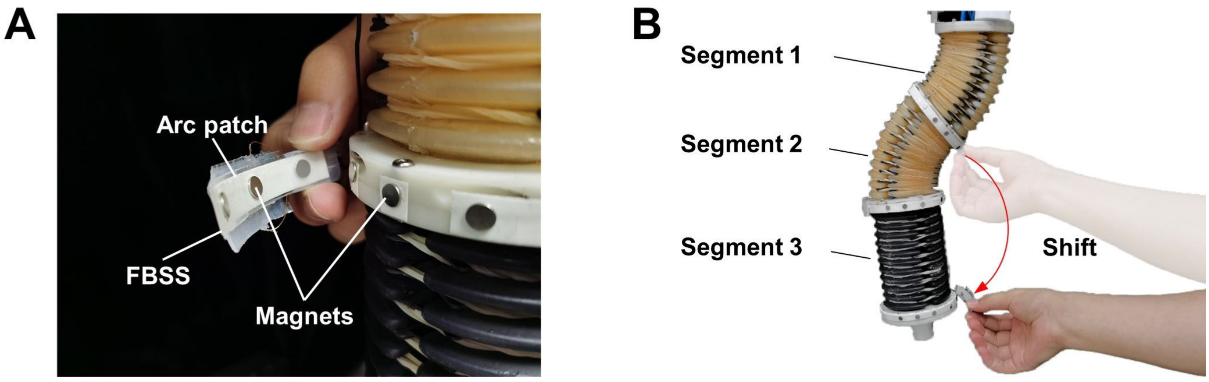

Supplementary Figure 13: Schematic diagram of quickly switching the position of a sensor on a soft manipulator.

(A) The photo of the FBSS integrated with the soft manipulator with small magnets. **(B)** The position of the FBSS can be quickly shifted with one hand.

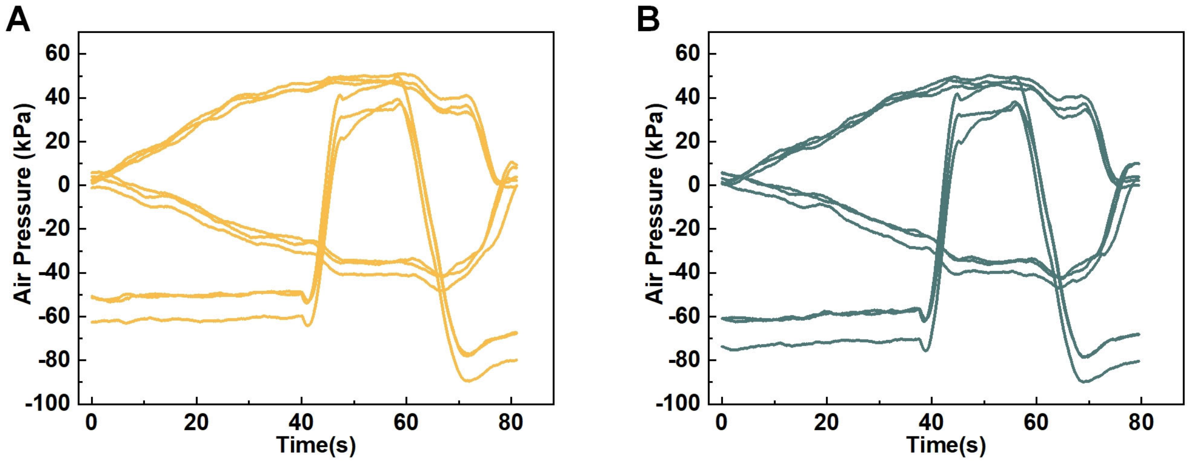

Supplementary Figure 14: Comparing the driven air pressures during teaching and repeating while taking a throat swab.

(A) Driven air pressure over time during teaching and **(B)** the same chart while repeating.

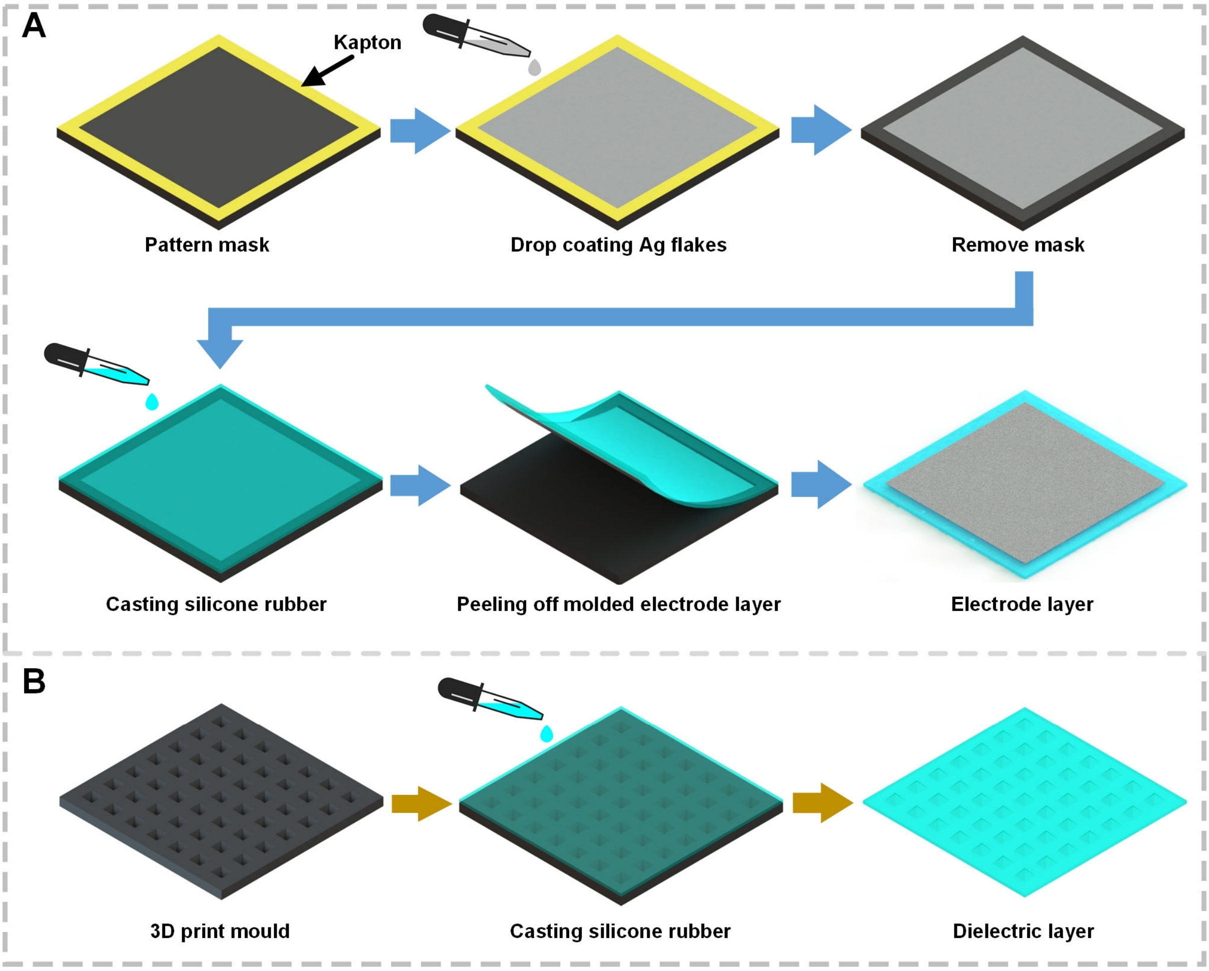

Supplementary Figure 15: Fabrication processes of the flexible electrode layer and the flexible dielectric layer.

(A) Fabrication of the flexible electrode layer. **(B)** Fabrication of the flexible dielectric layer.

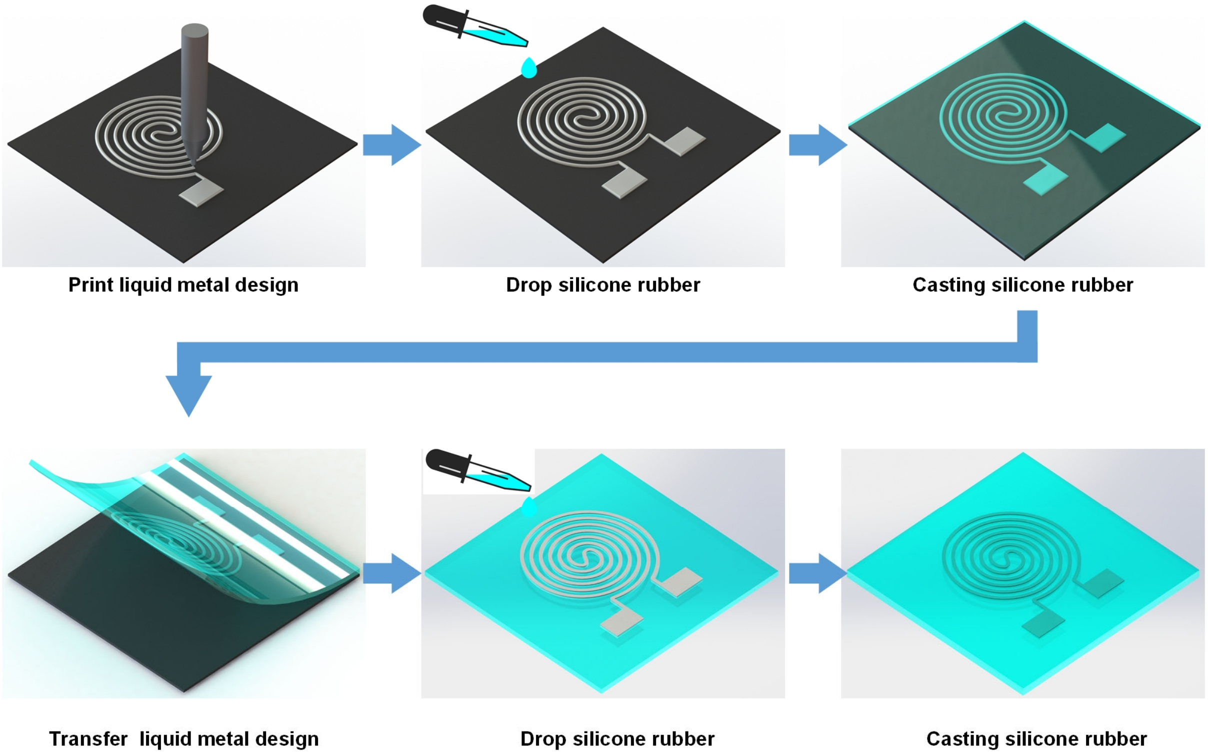

Supplementary Figure 16: Fabrication process of the liquid metal patch.

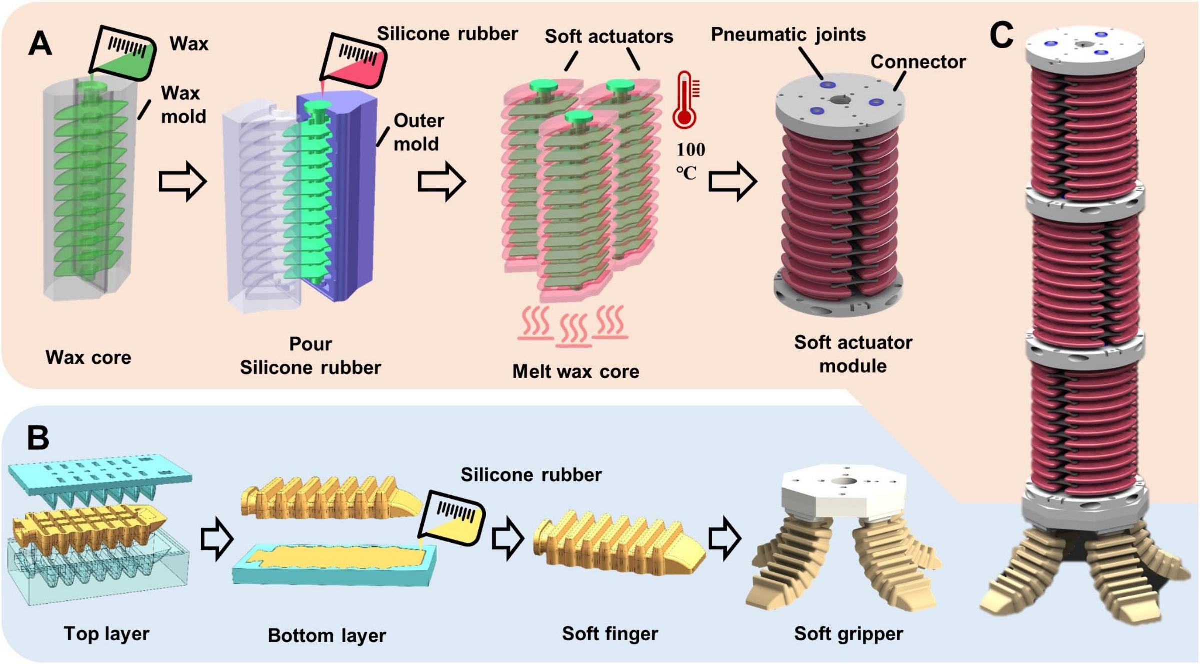

Supplementary Figure 17: Fabrication process of the soft manipulator.

(A) Fabrication of the soft actuator module. First, a wax core was fabricated with soft wax molds. Next, the soft actuator was fabricated with an outer mold. The soft actuator module was completed by adding universal joints and pneumatic connectors. **(B)** Fabrication of the four-fingered soft gripper. The fingers were fabricated by first molding a top layer and then sealed on a bottom layer. **(C)** Assembling the soft manipulator with a soft actuator module and soft gripper.

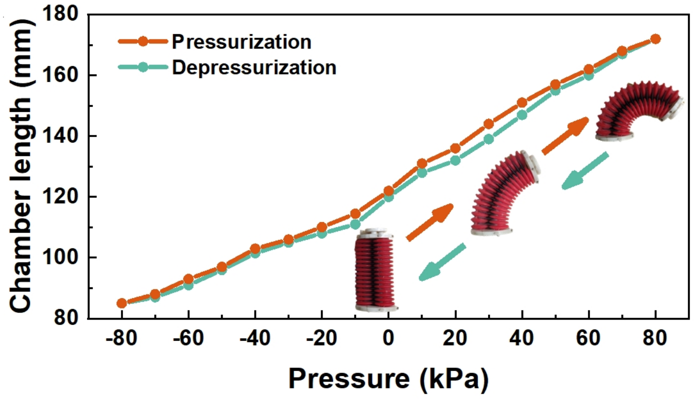

Supplementary Figure 18: Chamber lengths of the bending segments with different pressure.

Chamber lengths of the bending segments as a hysteretic function of the actuation pressure (-80 kPa to 80 kPa) in the pressurization (orange) and depressurization (green).

Supplementary Table 1. Algorithm for touchless human-soft manipulator single segmentary interaction.

Algorithm 1. Touchless human-soft manipulator single segmentary interaction

---

Input: The FBSS feedback signal V.

Output: The reaction of a single segment to avoid contact with human hand.

Set: Orientation of the FBSS.

The measured voltage V, the maximum voltage output V_{max}, the initial length of the segment l_{init}.

if The FBSS is mounted on the side of the soft segment

if $V > 0.6V_{\text{max}}$ // Hand approaching

Moveto($\pi/6l_{\text{init}}$, $\pi/6$, $\pi/2$) // Segment bending

endif

elseif The FBSS is mounted on the top of the soft segment

if $V > 0.6V_{\text{max}}$ // Hand approaching

Chamber pressures $\{p_1, p_2, p_3\}$←{-80kPa, -80kPa, -80kPa} // Segment shortening

end if

function Moveto(κ, θ, φ)

Chamber pressures $\{p_1, p_2, p_3\}$←$f_{\text{inv}}(\kappa, \theta, \varphi)$

Execute actuation with pressures p_{ij}, and the soft manipulator moves to position.

end function

Supplementary Table 2. Algorithm for human-soft manipulator interaction in two-dimensional space.

Algorithm 2. Touchless human-soft manipulator interaction with 2-DoFs

Input: The FBSS feedback signal (V, R).

Output: The end effector of the soft manipulator reaches the position targeted by a human hand.

Set: Arc parameters of a single segment of the soft manipulator, (κ_0, θ_0, φ_0).

Parameters of the hyperbolic tangent function, (k_1, k_2).

The initial step length, h_{init}.

The measured voltage V, the maximum voltage output $V_{\max}$, the initial voltage output V_{init}

while $R < 5\Omega$

The normalized FBSS voltage signal $S_{\mathrm{out}} \leftarrow \frac{V - V_{\mathrm{init}}}{V_{\max} - V_{\mathrm{init}}}$

Step length $\theta_{\mathrm{h}} \leftarrow h_{\mathrm{init}} \cdot \tanh\left(k_1^{\left(1-\frac{1}{k_2 S_{\mathrm{out}}}\right)} \right)$

Arc parameters of the soft manipulator $\theta \leftarrow \theta_0 + \theta_{\mathrm{h}}$

Bendto(θ)

$\theta_0 \leftarrow \theta$

end while

Execute actuation of the soft gripper.

function *Bendto(θ)*

for i=1,2,3

Chamber pressures $\{p_{i1}, p_{i2}, p_{i3}\} \leftarrow f_{\mathrm{inv}}(\kappa_0, \theta, \varphi_0)$

end for

Execute actuation with pressures p_{ij} and the soft manipulator moves into position.

end function

Supplementary Table 3. Algorithm for human-soft manipulator interaction in three-dimensional space.

Algorithm 3. Touchless human-soft manipulator interaction with 3-DoFs

Input: The FBSS feedback signal $\{V^i, R^i, i = 1, 2\}$.

Output: The end effector of the soft manipulator reaches the position targeted by a human hand.

Set: Arc parameters of a single segment of the soft manipulator, $(\kappa_0, \theta_0, \varphi_0)$.

Parameters of the hyperbolic tangent function, $\left\{k_1^i, k_2^i, i = 1, 2\right\}$.

The initial step length, h_{init}.

The measured voltage V^i, the maximum voltage output V_{max}^i, the initial voltage output V_{init}^i

while $R^2 < 8\Omega$

for i =1, 2

The normalized FBSS voltage signal $S_{out} \leftarrow \frac{V^i - V_{init}^i}{V_{max}^i - V_{init}^i}$

Step length $\theta_h^i \leftarrow h_{init} \cdot \tanh\left(k_1^{i\left(1 - \frac{1}{k_2 S_{out}}\right)}\right)$

Arc parameters of the soft manipulator $\theta^i \leftarrow \theta_0 + \theta_h$

Bendto(θ^i,*i*)

$\theta_0 \leftarrow \theta^i$

end for

end while

Execute actuation of the soft gripper.

function *Bendto(θ,i)*

if $i = 1$

Chamber pressures $\{p_{i1}, p_{i2}, p_{i3}\} \leftarrow f_{\text{inv}}(\kappa_0, \theta, \varphi_0)$

$i \leftarrow i + 1$

Chamber pressures $\{p_{i1}, p_{i2}, p_{i3}\} \leftarrow f_{\text{inv}}(\kappa_0, \theta, \varphi_0)$

else

$i \leftarrow i + 1$

Chamber pressures $\{p_{i1}, p_{i2}, p_{i3}\} \leftarrow f_{\text{inv}}(\kappa_0, \theta, \varphi_0)$

end if

Execute actuation with pressures p_{ij} and the soft manipulator moves into position.

end function

Supplementary Table 4. Comparison of bimodal sensors

Materials	Type	Stimuli	Deformability	Dual tactile/Touchless modes	Material identification	Touchless interactive media	Signal distinguishability	Ref.
Liquid metal electrode/hollow pillar-structured dielectric	Triboelectric/ Capacitive	Pressure/ Strain	Bend	No	No	/	Yes	[1]
Flexible organic tribotronic transistor (FOTT)	Triboelectric	Pressure/ Magnetic field	Bend	Yes	No	Magnetic field	No	[2]
Micro-pyramid NdFeB/PDMS	GMR effect	Pressure/ Magnetic field	Bend	Yes	No	Magnetic field	Yes	[3]
Magnetic tilt micropillar array-structured PDMS membrane	Capacitive	Pressure/ Magnetic field	Bend	Yes	No	Magnetic field	Yes	[4]
**AgNWs on PDMS /Liquid metal**	**Triboelectric / Resistance**	**Pressure/ Distance**	**Stretch Bend**	**Yes**	**Yes**	**Bare hand**	**Yes**	**This work**

Supplementary References

1. Zhang, C. *et al.* A stretchable dual-mode sensor array for multifunctional robotic electronic skin. *Nano Energy* **62**, 164–170 (2019).
2. Zhao, J. *et al.* Flexible Organic Tribotronic Transistor for Pressure and Magnetic Sensing. *ACS Nano* **11**, 11566–11573 (2017).
3. Ge, J. *et al.* A bimodal soft electronic skin for tactile and touchless interaction in real time. *Nat. Commun.* **10**, 1–10 (2019).
4. Zhou, Q. *et al.* Tilted magnetic micropillars enabled dual-mode sensor for tactile/touchless perceptions. *Nano Energy* **78**, (2020).
