# Supplementary material for: Touchless interactive teaching of soft robots through flexible bimodal sensory interfaces
Source: Nat Commun. 2022 Aug 26;13:5030. doi: 10.1038/s41467-022-32702-5 (PMC9412806; doi:10.1038/s41467-022-32702-5)
Supplement: Supplementary file 3 — Description of Additional Supplementary Files [file 41467_2022_32702_MOESM3_ESM.docx]

**Supplementary Movie Legends:**

**File Name:** Supplementary Movie 1.

**Description:** The FBSS detecting a human finger approaching and pressing it.

**File Name:** Supplementary Movie 2.

**Description:** The FBSS detecting a tennis ball falling.

**File Name:** Supplementary Movie 3.

**Description:** The FBSS detecting a feather falling.

**File Name:** Supplementary Movie 4.

**Description:** A human finger controls LEDs via the FBSS.

**File Name:** Supplementary Movie 5.

**Description:** A human user controlling the soft manipulator via the FBSS.

**File Name:** Supplementary Movie 6.

**Description:** A self-reacting soft origami robot with the FBSS detecting and grasping a toy bug in sand.

**File Name:** Supplementary Movie 7.

**Description:** The soft gripper detecting and grasping a plastic cylinder with the FBSS.

**File Name:** Supplementary Movie 8.

**Description:** The interactive teaching and repeating processes of the soft manipulator with variable steps.

**File Name:** Supplementary Movie 9.

**Description:** Interactively teaching and repeating to grasp objects in different positions.

**File Name:** Supplementary Movie 10.

**Description:** The soft manipulator teaching process replayed at different speeds.

**File Name:** Supplementary Movie 11.

**Description:** Interactively teaching and repeating while overcoming an obstacle.

**File Name:** Supplementary Movie 12.

**Description:** Interactively teaching and repeating in three dimensions (front view).

**File Name:** Supplementary Movie 13.

**Description:** Interactively teaching and repeating in three dimensions (side view).

**File Name:** Supplementary Movie 14.

**Description:** Interactively teaching and repeating to grasp objects in different positions in three dimensions.

**File Name:** Supplementary Movie 15.

**Description:** Interactively teaching and repeating multiple motions by switching the positions of a FBSS.

**File Name:** Supplementary Movie 16.

**Description:** Interactively teaching two-dimensional movements using the "shifting sensors and teaching" method.

**File Name:** Supplementary Movie 17.

**Description:** Interactively teaching three-dimensional movements using the "shifting sensors and teaching" method.

**File Name:** Supplementary Movie 18.

**Description:** Repeating three-dimensional movements using the "shifting sensors and teaching" method.

**File Name:** Supplementary Movie 19.

**Description:** Interactively teaching and repeating to complete a pen-and-paper maze.

**File Name:** Supplementary Movie 20.

**Description:** Interactively teaching and repeating to take a throat swab.

**File Name:** Supplementary Movie 21.

**Description:** Interactively teaching of the soft manipulator to cross the obstacle and grasp a flower.

**File Name:** Supplementary Movie 22.

**Description:** Repeating of the soft manipulator to cross the obstacle and grasp a flower.
